# Loteprednol etabonate ophthalmic suspension 0.5 %: efficacy and safety for postoperative anti-inflammatory use

**DOI:** 10.1007/s10792-012-9589-2

**Published:** 2012-06-16

**Authors:** Michael Amon, Massimo Busin

**Affiliations:** 1 Department of Ophthalmology, Hospital of the Brothers of Charity, Johannes von Gott Platz 1, 1020 Vienna, Austria; 2 Villa Igea Hospital, Forlì, Italy

**Keywords:** Cataract surgery, Intraocular pressure, Loteprednol etabonate suspension, Ocular surgery, Postoperative ocular inflammation, Topical corticosteroids

## Abstract

Topical corticosteroids are routinely used as postoperative ocular anti-inflammatory drugs; however, adverse effects such as increased intraocular pressure (IOP) are observed with their use. While older corticosteroids such as dexamethasone and prednisolone acetate offer good anti-inflammatory efficacy, clinically significant increases in IOP (≥10 mmHg) are often associated with their use. Loteprednol etabonate, a novel C-20 ester-based corticosteroid, was retrometabolically designed to offer potent anti-inflammatory efficacy but with decreased impact on IOP. After exerting its therapeutic effects on the site of action, loteprednol etabonate is rapidly converted to inactive metabolites, resulting in fewer adverse effects. Randomized controlled studies have demonstrated the clinical efficacy and safety of loteprednol etabonate ophthalmic suspension 0.5 % for the treatment of postoperative inflammation in post-cataract patients with few patients, if any, exhibiting clinically significant increases (≥10 mmHg) in IOP. Furthermore, safety studies demonstrated a minimal effect of loteprednol etabonate on IOP with long-term use or in steroid responders with a much lower propensity to increase IOP relative to prednisolone acetate or dexamethasone. The anti-inflammatory treatment effect of loteprednol etabonate appears to be similar to that of rimexolone and difluprednate with less impact on IOP compared to difluprednate, although confirmatory comparative studies are needed. The available clinical data suggest that loteprednol etabonate is an efficacious and safe corticosteroid for the treatment of postoperative inflammation.

## Background

Ocular inflammation is common after ophthalmic surgery, particularly after surgical removal of cataracts combined with intraocular lens (IOL) implantation. This inflammatory response includes the metabolism of arachidonic acid to prostaglandins and leukotrienes and the recruitment of neutrophils and macrophages to the site of surgical trauma [1]. This process eventually manifests as a mild iritis, corneal edema, and increased cells and proteins (flare) in the anterior chamber of the eye, accompanied by hyperalgesia [2]. While recent advances in surgical techniques (smaller incisions), more efficient phacoemulsifiers, and improved viscoelastics have improved cataract surgery outcomes, postoperative inflammation and pain remain a major source of discomfort for patients. If left untreated, postoperative inflammation can lead to suboptimal vision results or complications such as cystoid macular edema (CME) [1, 3–6]. As surgical techniques have improved, so has patient demand for excellent postoperative vision without postoperative complications.

Ocular inflammation following cataract surgery is managed by topical anti-inflammatory drugs such as non-steroidal anti-inflammatory drugs (NSAIDs) and/or corticosteroids. Both are effective in resolving postoperative inflammation and pain, increasing patient comfort, and decreasing the risk of complications [1, 7–11]. NSAIDs act through inhibition of cyclooxygenase enzymes, thereby blocking the production of prostaglandins [12]. Corticosteroids have a broader mechanism of action. They inhibit phospholipase A2 in the inflammatory cascade, which converts membrane phospholipids to arachidonic acid, thereby inhibiting the cyclooxygenase and lipoxygenase pathways and the formation of all eicosanoids. Corticosteroids suppress both the early (capillary dilation, increased vascular permeability, recruitment of leukocytes) and late (deposition of fibrin, proliferation of inflammatory cells and chemokines) phases of inflammation [13–16]. However, they are also associated with side-effects, including steroid-induced intraocular pressure (IOP) elevation, lowered resistance to infection, risk of cataract formation, and decreased wound healing [16–20]. Of these, increased IOP is the most significant side-effect for the post-cataract patient, and is thought to be due to structural and biochemical changes in the trabecular meshwork causing increased resistance to aqueous humor outflow [21]. Steroid-induced IOP elevation has been reported to occur in 18–36 % of patients, termed steroid responders, [15, 22] with risk factors including a history of glaucoma, a familial predisposition toward glaucoma, or high myopia [22, 23]. Older corticosteroids, such as prednisolone and dexamethasone, are associated with a greater impact on IOP compared to newer corticosteroids [24].

Recent research indicates that NSAIDs may have a synergistic effect with corticosteroids, particularly for the prevention of CME. In most instances, NSAIDs are used in combination with topical ocular corticosteroids [25]. It is therefore vital for ophthalmic surgeons to be able to provide patients with a corticosteroid option that offers high efficacy yet does not result in an increase of IOP to clinically significant levels.

**Fig. 1:**
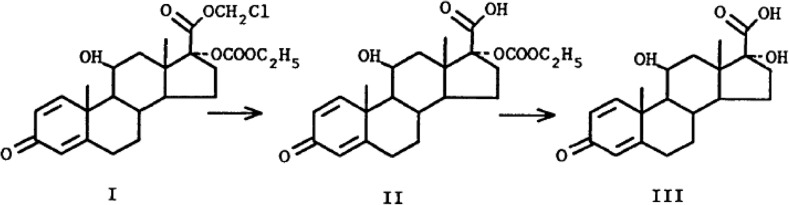
Loteprednol etabonate (*I*) and its inactive metabolites, Δ^1^ cortienic acid etabonate (*II*) and Δ^1^ cortienic acid (*III*)

Loteprednol etabonate is a novel corticosteroid produced by retrometabolic design. In retrometabolic drug design, an inactive and nontoxic metabolite of a reference compound is utilized as a starting point for conversion to a therapeutically active, metabolically labile compound [26]. Loteprednol etabonate was designed starting with Δ^1^ cortienic acid, an inactive metabolite of prednisolone. Structurally, loteprednol etabonate differs from prednisolone in that the ketone at the carbon-20 (C-20) position is replaced with a chloromethyl ester and the 17α-hydroxyl group is replaced with a carbonate moiety (Fig. 1). After exerting its effects, loteprednol etabonate is rapidly metabolized by tissue esterases to Δ^1^ cortienic acid etabonate and then to Δ^1^ cortienic acid, thereby limiting any potential adverse effects associated with its use. Preclinical studies demonstrated that loteprednol etabonate is highly lipophilic and has strong binding affinity to glucocorticoid receptors. Indeed, its lipophilicity was found to be 10 times greater while its binding affinity to the glucocorticoid receptor was found to be 4.3 times greater than that of dexamethasone [27, 28]. Approved in the United States in 1998 for the treatment and prevention of various steroid-responsive ocular inflammatory conditions as well as for the treatment of postoperative ocular inflammation, loteprednol etabonate has since received marketing approval in various countries across Europe, Latin America, the Middle East, North Africa, and Asia.

The objective of this paper was to review the available published clinical data on loteprednol etabonate suspension 0.5 % in the treatment of postoperative inflammation and pain, and to assess its efficacy and safety along with that of other corticosteroids formally studied and approved for the treatment of postoperative inflammation, namely, difluprednate and rimexolone. Publications on loteprednol etabonate were identified through MEDLINE searches (1950 onwards) using the terms loteprednol, postoperative pain, inflammation, cataract, and cataract surgery. In order to identify publications about other corticosteroids currently used in postoperative pain and inflammation, the terms rimexolone, difluprednate, prednisolone, dexamethasone, fluorometholone, cataract, cataract surgery, postoperative, and postsurgical inflammation were also searched. Only ophthalmic studies were included, and validity was assessed based on dosage form (topical only), indications for use, study endpoints, and year of publishing. The search was limited to English language, peer-reviewed primary studies and any reviews published in the last 5 years. Additional references were obtained by searching reference lists of identified articles. As no direct head-to-head studies comparing loteprednol etabonate to rimexolone or to difluprednate were found, insights on comparative safety and efficacy of loteprednol etabonate, rimexolone, and difluprednate were drawn from vehicle-controlled studies or from studies in which these newer corticosteroids were compared to older corticosteroids such as dexamethasone and/or prednisolone acetate.

## Loteprednol etabonate: efficacy and safety studies

The efficacy and safety of loteprednol etabonate ophthalmic suspension 0.5 % for the treatment of postoperative inflammation has been demonstrated by several studies over the last decade (Table 1). A double-masked, vehicle-controlled evaluation of the efficacy and safety of loteprednol etabonate for postoperative inflammation after cataract removal with IOP implantation was conducted in 1998 by the Loteprednol Etabonate Postoperative Study Group [2]. In this study, 203 patients with an anterior chamber inflammation (ACI) severity ≥3 (0–9 scale) on the day following cataract surgery were randomized to either loteprednol etabonate or vehicle administered four times daily in the operated eye for 14 days. Resolution of ACI, defined as ≤5 cells and none-to-trace flare, was observed in 55 % of patients in the loteprednol etabonate group and 28 % of patients in the vehicle group (27 % difference, *P* < 0.001) at the final visit. The rate for individual signs of cell and flare as well as supportive signs and symptoms of chemosis, erythema, bulbar injection, ciliary flush, pain, photophobia, tearing and discomfort, all favored the loteprednol etabonate group (*P* < 0.05). From a safety perspective, both treatment groups exhibited a mean decrease in IOP (1–2 mmHg) when compared with baseline. No patients in the loteprednol etabonate group versus a single patient in the vehicle group exhibited a clinically significant increase in IOP (≥10 mmHg). Stewart et al. [13] conducted an identical study to evaluate the efficacy and safety of loteprednol etabonate in controlling ACI. In this study, 227 post-cataract patients with ACI severity ≥3 (0–9 scale) on the day following surgery were randomized to loteprednol etabonate or vehicle. Resolution of ACI at the final visit was observed in 64 % of patients in the loteprednol etabonate and 29 % of patients in the vehicle groups (35 % difference, *P* < 0.001). Again, the resolution rate for cells and flare individually as well as supportive signs and symptoms of chemosis, erythema, bulbar injection, ciliary flush, pain, photophobia, tearing, and discomfort all favored the loteprednol etabonate group (*P* < 0.05). As in the first study, mean IOP decreased in both treatment groups relative to baseline by 1–2 mmHg. A clinically significant increase in IOP (≥10 mmHg) was observed in three patients in the loteprednol etabonate group. Comstock and Usner [29] further explored the efficacy of loteprednol etabonate in resolving pain and discomfort in these studies. The treatment effect for pain and discomfort was 31 and 43 %, respectively, for the first study and 24 and 30 %, respectively, for the second study. Analysis of pooled data indicated that the proportion of at-risk patients with resolution of pain at the final visit was 84 % for the loteprednol etabonate group and 56 % for the placebo group (*P* < 0.05). Similarly, resolution of discomfort at the final visit was 79 % for the loteprednol etabonate group and 42 % for the placebo group (*P* < 0.05).

**Table 1:** Studies demonstrating the efficacy and safety of loteprednol etabonate (LE) 0.5 % for postoperative inflammation

Study parameters	Stewart et al. [13]	LE postoperative study group 2 1998 [2]	Grigorian et al. [31]	Stewart [30]
Comparator	Vehicle	Vehicle	1 % (PA)	Fluorometholone acetate 0.1 % (FA)
No. of patients	203	227	20	30
Treatment duration (weeks)	2	2	4	2
Patients with resolution of ACI^a^ at final visit (%)	LE group—64	LE group—55	LE group—60	LE group—60
	Vehicle—29	Vehicle—28	PA group—50	FA group—100
Mean IOP at final visit	Mean decrease in IOP of 1–2 mmHg for both treatment groups	Mean decrease in IOP of 1–2 mmHg for both treatment groups	LE group—12 ± 3 mmHg	Not reported
			PA group—16 ± 1 mmHg	
Clinically significant increases in IOP (≥10 mmHg)	*n* = 3 for loteprednol etabonate; *n* = 0 for vehicle	*n* = 0 for loteprednol etabonate; *n* = 1 for vehicle	Not reported	Not reported

*ACI* anterior chamber inflammation, *IOP* intraocular pressure, *LE* loteprednol etabonate, *PA* prednisolone acetate, *FA* fluorometholone acetate

^a^Resolution of ACI defined as anterior chamber cell count <5 and flare Grade 0 (none)

Three small prospective studies further compared the efficacy and/or safety of loteprednol etabonate with that of other corticosteroids. Stewart [30] compared the efficacy and safety of loteprednol etabonate 0.5 % and fluorometholone acetate 0.1 % in the treatment of postoperative inflammation. A total of 30 post-cataract patients were enrolled in this randomized, double-masked, parallel-group study. All patients instilled a single drop of the assigned study medication four times daily for 14 days. At the final visit, no statistically significant differences in flare, anterior segment cell, or conjunctival hyperemia were observed between the two treatment groups. No significant adverse events were observed in either group. Grigorian et al. [31] compared the efficacy and safety of loteprednol etabonate and prednisolone acetate 1 %, in the treatment of postoperative inflammation following cataract surgery. Twenty patients were randomly assigned to loteprednol etabonate or prednisolone, instilled four times daily for the first week, tapering to once daily by week 4, in this randomized double-masked study. Patients from both groups achieved a similar resolution of postoperative inflammation (conjunctival hyperemia, corneal edema, aqueous cells, flare), with 60 % of patients in the loteprednol etabonate group and 50 % of patients in the prednisolone group achieving significant resolution of inflammation by the final visit. Despite the study’s small sample size, treatment with loteprednol etabonate had less effect on IOP elevation than prednisolone. The mean (SD) IOP on the final visit was 12 (3) mmHg in the loteprednol etabonate group compared with 16 (1) mmHg in the prednisolone group. Çoban and Kocak [32] also compared the safety of loteprednol etabonate 0.5 % and prednisolone acetate 1 % in 40 patients after uncomplicated phacoemulsification surgery. Treatments were administered five times daily from the first day postoperatively, and patients were evaluated at 1 day, 1 week, and 1 month thereafter. At all postoperative visits, the mean IOP was lower in the loteprednol etabonate group than in the prednisolone group. The authors concluded that loteprednol etabonate 0.5 % use after cataract surgery is associated with a smaller increase in IOP than prednisolone use.

As indicated above, in most instances, topical corticosteroids are used in combination with NSAIDs for the treatment of pain and postoperative inflammation. Macri et al. [25] compared the use of loteprednol etabonate 0.5 % alone, loteprednol etabonate 0.5 % in combination with indomethacin 0.1 %, and dexamethasone disodium phosphate 0.15 % in the treatment of postoperative inflammation following uncomplicated cataract surgery. Patients were divided into three groups, the first of which included 79 patients administered indomethacin 0.1 % from 3 days before to 2 weeks after surgery and loteprednol etabonate for 4 weeks after surgery. The second and third groups comprised 81 patients treated with loteprednol etabonate 0.5 % for 4 weeks and 78 patients treated with dexamethasone for 4 weeks, respectively. All three therapeutic regimens were effective in preventing postoperative ocular inflammation at postoperative weeks 1 and 4 with very mild ACI and very low corneal fluorescein staining in all 3 groups. Two cases of IOP elevation (both in the dexamethasone group) and three cases of CME (loteprednol etabonate group = 1, and dexamethasone group = 2) were observed. The authors concluded that there was better control of IOP and prevention of CME in the combination group but that larger confirmatory studies were needed.

## Loteprednol etabonate: additional safety studies

The above studies establish the efficacy and/or safety of loteprednol etabonate 0.5 % for the treatment of postoperative inflammation in prospective, randomized, double-masked studies. Additional published studies provide insight into the safety of long-term treatment with loteprednol etabonate and safety of loteprednol etabonate in steroid responders.

Howes et al. [33] studied the systemic pharmacokinetics, systemic effects, and IOP effects of loteprednol etabonate 0.5 % after chronic ocular administration in a double-masked study. Healthy individuals aged 19–44 years were randomized to receive either loteprednol etabonate (*n* = 10) or vehicle (*n* = 4) instilled in each eye every 2 h while awake (8 times a day) on days 0 and 1 and four times daily on days 2 through to 42. Blood samples were collected at 0, 15, 30, 60, and 120 min after the first and eighth doses on day 0 and after the fourth dose on day 42, and once on days 7, 14, and 28. Plasma levels of loteprednol etabonate and Δ^1^ cortienic acid etabonate were below the level of quantitation (1 ng/mL) in all samples collected, while cortisol levels were all within normal range indicating a lack of hypothalamic–pituitary–adrenal axis suppression. None of the patients exhibited a clinically significant increase in IOP (≥10 mmHg) over the 6 week study period. Holland et al. [34] compared the effects of a combination of loteprednol etabonate and tobramycin (LE/T) with that of dexamethasone and tobramycin (DM/T) on IOP in healthy volunteers over a 4 week treatment period. A total of 306 volunteers were randomized to receive either LE/T or DM/T administered every 4 h for 28 days. Clinically significant increases in IOP (≥10 mmHg) were observed in three (1.95 %) subjects treated with LE/T compared with 11 (7.48 %) subjects treated with DM/T (*P* = 0.028). Finally, Novack et al. [35] assessed the long-term safety of loteprednol etabonate across all development studies of loteprednol etabonate (0.2 and 0.5 % suspension) in a large retrospective analysis. These development studies included the use of loteprednol etabonate for postoperative inflammation as well as other ocular inflammatory conditions (seasonal allergic conjunctivitis, giant papillary conjunctivitis, and uveitis). Data from 1,648 patients treated with loteprednol for ≥28 days was reviewed. The proportion of patients exhibiting clinically significant increases in IOP (≥10 mmHg) was 0.5, 1.7, and 6.7 % with vehicle, loteprednol etabonate, and prednisolone acetate 1 % (used as a comparator in some studies), respectively. Excluding patients that continued to wear contact lenses (allowed in giant papillary conjunctivitis studies), the proportions of patients showing clinically significant increases in IOP were 1.0, 0.6, and 6.7 % for vehicle, loteprednol etabonate, and prednisolone acetate 1 %, respectively. In studies with loteprednol etabonate 0.5 %, the proportion of patients with a clinically significant increase in IOP was 2.1 % if patients wearing contact lenses were included and 0.8 % if these patients were excluded.

In addition to the above studies on the safety of loteprednol etabonate with long-term use, two studies in steroid responders support the relative lack of impact of loteprednol etabonate on IOP. In a retrospective review, Holland et al. [36] evaluated data from 30 post-penetrating keratoplasty and post-keratolimbal allograft patients who, after experiencing increased IOP to ≥21 mmHg, were switched from prednisolone acetate 1 % to loteprednol etabonate 0.5 %. Results showed a mean (SE) reduction of IOP from 31.1 (1.13) mmHg on prednisolone acetate to 18.2 (1.37) mmHg on loteprednol etabonate (*P* < 0.001) and no allograft rejection. Bartlett and colleagues examined the safety of loteprednol etabonate in a crossover study in 19 known steroid responders. Subjects received either loteprednol etabonate or prednisolone acetate 1.0 % for 42 days followed by a washout period of 14 days prior to being crossed over to the other treatment [37]. During treatment with loteprednol etabonate, IOPs were within the normal range, with a mean IOP elevation of 4.1 mmHg over the 42 day period (*NS* vs baseline). In contrast, the subjects’ IOPs were significantly greater compared to baseline during treatment with prednisolone acetate; mean IOP elevations of 5.9, 7.7, and 9.0 mmHg were observed by days 14, 28, and 42, respectively (*P* < 0.05 for all).

Taken together, the available clinical efficacy and safety studies of loteprednol etabonate 0.5 % for the treatment of postoperative inflammation, combined with safety studies on the long-term use of loteprednol etabonate or use of loteprednol etabonate in steroid responders suggest that loteprednol etabonate is a potent and safe topical corticosteroid with a low propensity to increase IOP relative to older corticosteroids such as prednisolone acetate or dexamethasone.

## Loteprednol etabonate: comparison with rimexolone

Rimexolone is a C-20 ketone steroid similar to prednisolone with the 17α-hydroxyl group replaced with a methyl group and an additional methyl group at the C16 position [38]. While it has been granted regulatory approval in the United States, rimexolone has not received approval in all countries in the European Union. Bron et al. [39] assessed the safety and efficacy of rimexolone 1 % ophthalmic suspension compared with placebo for reducing postoperative inflammation after cataract surgery and IOL implantation. In this study, 182 post-cataract patients were randomized to rimexolone 1 % or placebo instilled four times daily for 14 days postoperatively. As was the case in studies with loteprednol etabonate, inclusion criteria included a Grade ≥3 (0–9 scale) for the sum of cells and flare combined on the day following cataract surgery. By the final visit, the proportion of patients with resolution of inflammation (≤5 cells and none-to-trace flare) was 59.7 and 27.6 % for the rimexolone and placebo group, respectively (32 % difference; *P* < 0.0001). Supportive measures of ocular discomfort, corneal edema, bulbar conjunctival erythema, anterior vitreous reaction and the physician’s impression all favored treatment with rimexolone (*P* < 0.05). The authors reported no perceptible change in IOP in either group at any visit but indicated that the study was not designed to show differences in IOP. A similar study was conducted by Assil et al. [7]. In this study, 197 patients with combined cell and flare severity ≥3 for cells and flare combined (0–9 scale) 24 h after cataract surgery were randomized to rimexolone 1 % or placebo four times daily for 14 days. Consistent with the study by Bron et al., 59.7 % of patients in the rimexolone group compared to 32.1 % of patients in the placebo group had their inflammation resolved by the final visit (27.6 % difference, *P* < 0.001). Secondary measures of bulbar conjunctival erythema, and the physician’s follow-up impression also favored rimexolone treatment compared to placebo treatment (*P* < 0.05) although there was no between-group difference in the presence of ciliary flush or anterior vitreous reaction. Mean IOP decreased relative to baseline (postoperative day 1) in both treatment groups, although two patients in each treatment group experienced a clinically significant increase in IOP (≥10 mmHg). Based on the treatment effects noted in these studies, the clinical efficacy observed with rimexolone appears similar to that observed in vehicle-controlled studies with loteprednol etabonate.

Several published studies compared the clinical efficacy and safety of rimexolone 1 % to prednisolone acetate 1 %. Kavuncu et al. [40] compared the efficacy of rimexolone to prednisolone, administered four times daily for 15 days postoperatively for the treatment of postoperative ocular inflammation in 80 post-cataract patients (baseline inflammation severity not specified). Both treatments were effective in reducing postoperative inflammation, with no between-group differences in anterior chamber cell count or flare severity at any postoperative visit (days 1, 3, 8, 15, 18). However, conjunctival hyperemia was worse in the rimexolone group on days 1 and 3 (*P* < 0.05), while corneal edema was worse in the prednisolone group on day 8 (*P* < 0.05). There were also no between-group differences in IOP, with mean (SD) final visit IOPs of 12.96 (3.2) mmHg and 11.65 (2.86) mmHg, respectively. Yaylali et al. [41] also evaluated the anti-inflammatory efficacy of rimexolone with that of prednisolone acetate 1 %, both administered four times daily for 15 days, in patients (*n* = 48) undergoing cataract extraction by phacoemulsification. There was no difference between treatment groups in mean inflammation scores (aqueous cell, flare, and conjunctival hyperemia individually) at postoperative follow-up visits except for cells at postoperative day 3 which were lower in the prednisolone group. Although mean IOP decreased in both groups relative to postoperative day 1, a significant difference between treatments was found at postoperative day 3, with mean (SD) IOPs of 10.9 (1.3) mmHg and 11.9 (1.9) mmHg in the rimexolone and prednisolone groups, respectively. There were no differences between treatments in mean IOP at days 7 or 15. Furthermore, Hirneiss et al. [42] compared the clinical efficacy and safety of rimexolone with that of prednisolone and ketorolac tromethamine in 45 patients after cataract extraction. There was no difference between treatments in control of aqueous cells, but prednisolone was more effective than rimexolone in controlling flare and conjunctival hyperemia. Notably, one patient from the prednisolone group was discontinued for a marked early increase in IOP.

Finally, Leibowitz et al. [38] studied the IOP-increasing potential of rimexolone 1.0 % with that of fluorometholone alcohol 0.1 % in a double-masked, two-way crossover study of 45 otherwise healthy steroid responders. Following verification of steroid responder status with either dexamethasone or prednisolone acetate, subjects were randomized to either rimexolone or fluorometholone administered every 2 h for 2 days followed by four times daily for 40 days. On completion of the 6 week study duration or on exhibiting an increase in IOP of ≥10 mmHg, the study treatment was stopped; subjects completed a 1 month washout and then received the alternate treatment for another 6 weeks. There was no significant difference between rimexolone and fluorometholone in the number of subjects demonstrating an increase in IOP of ≥10 mmHg or in the number of weeks required to achieve a 10 mmHg increase. Rimexolone treatment resulted in a mean increase in IOP of 7.5 mmHg in patients previously observed to have a mean increase of 11.8 mmHg with dexamethasone (*P* = 0.001), and a mean increase of 6.2 mmHg in patients previously observed to have a mean increase of 12.1 mmHg with prednisolone acetate (*P* < 0.001). As noted above, a similar cross-over study with loteprednol etabonate resulted in a mean IOP elevation of 4.1 mmHg over a six-week period (*NS* vs baseline) compared to 9.0 mmHg for prednisolone acetate (*P* < 0.05 vs baseline) [37]. Table 2 compares the efficacy and safety parameters evaluated in the above-mentioned studies on rimexolone.

**Table 2:** Studies demonstrating the efficacy and safety of rimexolone 1 % for postoperative inflammation

Study parameters	Bron et al. [39]	Assil et al. [7]	Kavuncu et al. [40]	Yaylali et al. [41]	Hirneiss et al. [42]
Comparator	Placebo	Placebo	PA	PA	PA and ketorolac
No. of patients	182	197	80	48	45
Treatment duration (weeks)	2	Up to 2	2	2	4
Patients with resolution of ACI^a^ at final visit ( %)	Rimexolone—60 %	Rimexolone—60 %	Not reported^b^	Not reported^b^	Not reported^b^
	Vehicle—28 %	Placebo—32 %			
Mean IOP at final visit	No perceptible changes in IOP	Mean decrease in IOP in both treatment groups compared to baseline	Rimexolone—13.0 ± 3.2 mmHg	Rimexolone—11.6 ± 1.4 mmHg	Rimexolone—13.25 mmHg
					PA group—14.60 mmHg
			PA group—11.7 ± 2.9 mmHg	PA group—10.8 ± 1.3 mmHg	Ketorolac group—13.73 mmHg
Clinically significant increases in IOP (≥ 10 mmHg)	Not reported	*n* = 2 for rimexolone;	Not reported	Not reported	Not reported
		*n* = 2 for placebo			

*ACI* anterior chamber inflammation, *IOP* intraocular pressure, *PA* prednisolone acetate

^a^Resolution of ACI defined as anterior chamber cell count <5 and flare Grade 0 (none)

^b^The study did not report the percentages of patients with resolution of ACI; instead, mean cell and flare at study visits were reported

In summary, vehicle-controlled studies with rimexolone suggested similar treatment effects compared to loteprednol etabonate for the control of postoperative inflammation with minimal IOP impact. However, contrary to comparative studies with loteprednol etabonate, studies comparing rimexolone with prednisolone acetate suggest rimexolone’s clinical efficacy may not be as robust as that of prednisolone.

## Loteprednol etabonate: comparison with difluprednate

**Fig. 2:**
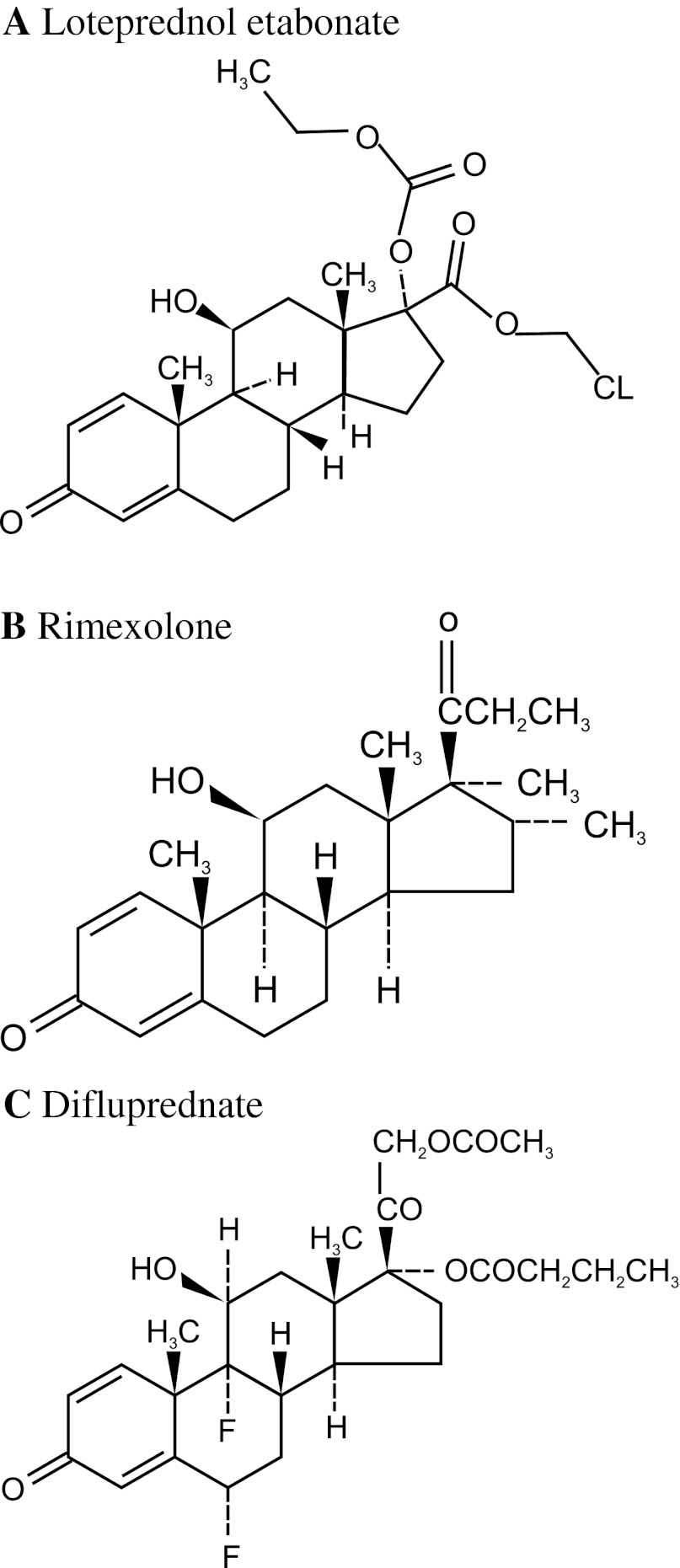
Structures of loteprednol etabonate (**a**), rimexolone (**b**) and difluprednate (**c**)

Difluprednate ophthalmic emulsion, 0.05 %, has also been approved for postoperative anti-inflammatory use in the United States. Like loteprednol etabonate, difluprednate is a derivative of prednisolone. Structural modifications include the addition of fluorine atoms at both the C-6 and C-9 positions, a butyrate ester at the C-17 position and acetate ester at the C-21 position (Fig. 2) [43]. However, difluprednate retains the C-20 ketone moiety of prednisolone. Korenfeld et al. [8] evaluated the safety and efficacy of difluprednate 0.05 % versus placebo for postoperative inflammation in two identical double-masked, placebo-controlled studies. Patients (*n* = 438) with anterior chamber cell grade ≥2 one day after ocular surgery were randomized to difluprednate twice daily, difluprednate four times daily or placebo (*n* = 110 in each study) for 14 days followed by a 14 day tapering period. Primary assessment included the resolution of anterior chamber cells, proportion of patients with clinical response, and absence of pain/discomfort. For comparative purposes, only the proportion of patients with clinical response (defined as ≤5 cells and no flare) is summarized here. By day 15, clinical response was observed in 72.7 % of patients receiving difluprednate twice daily and 71.0 % of patients receiving difluprednate four times daily compared to approximately 27 % of patients receiving placebo (difference ~45 %, *P* < 0.0001 vs placebo). Proportions for pain-free patients and secondary measures of photophobia, chemosis, corneal edema, and conjunctival injection were all significantly better in the difluprednate groups compared to placebo groups. In terms of safety, mean IOP remained within normal range in all treatment groups; however, three patients (3 %) in each of the difluprednate treatment groups experienced clinically significant increases in IOP compared with two patients (1 %) in the placebo group. Smith et al. [44] studied the clinical efficacy of difluprednate administered twice daily for managing ocular inflammation and pain following cataract surgery. Patients (*n* = 121) were randomized to receive either difluprednate or placebo twice daily for 16 days; this was followed by a 14 day tapering period. This study differed from previous studies in that dosing was initiated 24 h before ocular surgery. Resolution of cells (≤5 cells) and Grade 0 flare was observed in 74.7 % of difluprednate patients compared to 42.5 % of placebo patients (difference = 32 %; *P* = 0.0006). Significant differences were also observed between the two groups in the proportions of patients that were free of ocular pain and discomfort (difference = 34.6 %; *P* = 0.0004). As in the study by Korenfeld et al. [8], three subjects (3.7 %) in the difluprednate group had a clinically significant increase in IOP (≥10 mmHg). Further details of the above-mentioned trials are provided in Table 3.

**Table 3:** Studies demonstrating the efficacy and safety of difluprednate for postoperative inflammation

Study parameters	Korenfeld et al. [8]	Smith et al. [44]
Comparator	Placebo	Placebo
No. of patients	438	124
Treatment duration	15 days^a^	16 day treatment period^a^
Patients with a clinical response^b^ (%)	Difluprednate BID—72.7^c^	Difluprednate—74.7 %
	Difluprednate QID—71^c^	Placebo—42.5
Mean IOP at final visit	No significant changes from baseline for the difluprednate group reported	Not reported
Clinically significant increases in IOP (≥10 mmHg)	*n* = 3 for difluprednate BID;	*n* = 3 for difluprednate
	*n* = 3 for difluprednate QID;	
	*n* = 2 for placebo	

*ACI* anterior chamber inflammation, *BID* twice daily, *QID* 4 times daily, *IOP* intraocular pressure

^a^The treatment period was followed by a 2 week tapering period before treatment was stopped

^b^Clinical response defined as anterior chamber cell count <5 and flare Grade 0

^c^Prior to commencement of dose-tapering

In summary, while the above placebo-controlled studies suggest difluprednate has a similar treatment effect to loteprednol etabonate, the impact of difluprednate on IOP may be greater. Indeed, Cable [45] reported significant elevations of IOP in patients undergoing uncomplicated cataract surgery administered difluprednate twice daily following surgery. In this retrospective chart review of 100 consecutive patients, 5 % of patients responded with ocular hypertension. All patients had a history of open-angle glaucoma, but were not known steroids responders, and the average increase in IOP was 17.8 mmHg. The IOP increases were managed by the discontinuation of difluprednate, and the administration of topical glaucoma medication if required. The IOP returned to baseline in all patients in 1–2 days. In contrast, IOP of ≥ 10 mmHg was seen on average in 1.4 % of loteprednol etabonate treated patients in vehicle-controlled clinical studies and as few as 1.7 % of long-term users of loteprednol etabonate [35, 46].

**Fig. 3:**
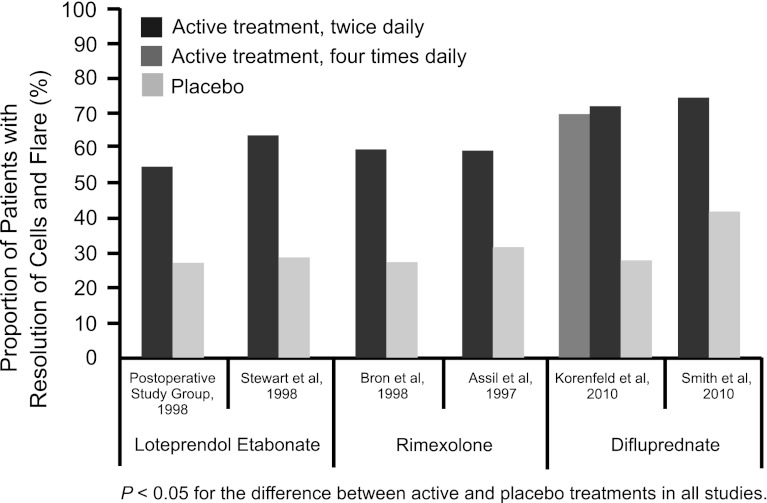
Resolution of cells and flare in clinical studies of loteprednol etabonate 0.5 %, rimexolone 1 %, and difluprednate 0.5 % for postoperative inflammation following uncomplicated cataract surgery. Resolution of cells and flare was defined as ≤5 cells and none-to-trace flare in loteprednol etabonate and rimexolone studies and <5 cells and no flare in difluprednate studies

Figure 3 compares the resolution of cells and flare in clinical studies of loteprednol etabonate 0.5 %, rimexolone 1 %, and difluprednate 0.5 % for postoperative inflammation following uncomplicated cataract surgery, based on vehicle-controlled studies.

## Conclusion

As evidenced by a comprehensive review of the data from published studies, loteprednol etabonate is effective in resolving anterior chamber cells and flare as well as in reducing postoperative pain and discomfort. Based on the available data, loteprednol etabonate offers efficacy similar to older corticosteroids such as prednisolone acetate, with a much-reduced effect on IOP, thereby presenting an improved safety profile as compared to these older compounds. Extensive searches through the available literature have demonstrated a lack of direct head-to-head studies comparing loteprednol etabonate to other newer corticosteroids (such as rimexolone and difluprednate) formally approved for this indication. In order to address this gap, we compared the relative safety and efficacy of loteprednol etabonate with these newer corticosteroids across vehicle-controlled studies and by examining data from studies in which these compounds were compared to older corticosteroids. Based on our results, loteprednol etabonate provides similar efficacy to rimexolone and difluprednate by offering similar rates of resolution of ocular inflammation. The use of loteprednol etabonate, however, seems to be associated with fewer clinically significant increases in IOP (≥10 mmHg), thereby reducing the risk of corticosteroid-induced ocular hypertension and eventual corticosteroid-induced glaucoma. While these results provide significant insights into the effects of these newer corticosteroids, high quality, active-controlled, randomized clinical trials between these compounds are needed to assess their comparative safety and efficacy.
